# Venoms as an adjunctive therapy for Parkinson’s disease: where are we now and where are we going?

**DOI:** 10.2144/fsoa-2020-0119

**Published:** 2020-11-30

**Authors:** Parisa Gazerani

**Affiliations:** 1 Laboratory of Molecular Pharmacology, Department of Health Science & Technology, Faculty of Medicine, Aalborg University, 9220 Aalborg East, Denmark

**Keywords:** animal toxins, bee venom, dopaminergic, lizard venom, neurological disease, Parkinson’s disease, scorpion venom, snake venom, therapeutics, venom

## Abstract

Neurodegenerative diseases, including Parkinson’s disease (PD), are increasing in the aging population. Crucially, neurodegeneration of dopaminergic neurons in PD is associated with chronic inflammation and glial activation. Besides this, bradykinesia, resting tremor, rigidity, sensory alteration, and cognitive and psychiatric impairments are also present in PD. Currently, no pharmacologically effective treatment alters the progression of the disease. Discovery and development of new treatment strategies remains a focus for ongoing investigations. For example, one approach is cell therapy to prevent dopaminergic neuronal loss or to slow PD progression. The neuroprotective role of a diverse range of natural products, including venoms from bees, scorpions, snakes and lizards, are also being tested in preclinical PD models and in humans. The main findings from recent studies that have investigated venoms as therapeutic options for PD are summarized in this special report.

## Lay abstract

Parkinson’s disease (PD) is a progressive disease of an aging brain that is characterized by tremors, stiffness, gradual loss of memory, depression, sleep problems and fatigue. Current PD drug treatments target these symptoms; however, new approaches are being investigated to slow down or prevent disease progression. One approach is to use venoms (e.g., bee venom or its components) to protect the brain from further damage caused by PD progression. This special report presents current scientific findings to support the future use of venoms as therapeutic options for PD.

## Parkinson’s disease

It is estimated that 22% of the population will be over 60 by 2050 [1]. Consequently, age-related diseases have attracted more attention because of their high impact on healthcare systems and their social and economic burden [2]. Hence, the negative impact of age-related disorders of the nervous system, including Parkinson’s disease (PD), continue to rise [3]. A ‘Parkinson pandemic’ [4], where up to 17 million people will be affected by this disorder is expected by 2040. This issue calls for focused planning, immediate and long-term actions and novel approaches to prevent and control this condition.

PD is characterized by progressive neurodegeneration in the substantia nigra pars compacta (SNpc), that leads to the loss of dopaminergic neurons [5]. Research focused on the molecular pathogenesis of PD demonstrates a role for protein aggregates [6]. Specific α-synuclein conformations within the CNS have been identified to cause direct or, via propagation of Lewy pathology, indirect neuronal damage [6]. Other mechanisms that include altered calcium homeostasis, axonal transport, chronic neuroinflammation and glia activation, mitochondrial dysfunction and oxidative stress have been proposed [3,7,8]. PD is characterized by motor dysfunction including bradykinesia, resting tremor and rigidity [9]. Neuropsychiatric disturbances, are also common in PD [10]. Sensory alterations (e.g., sense of smell and touch) in PD patients normally appear at earlier stages of the disease [11,12]. Risk factors for disease progression such as advanced age, genetic predisposition [13,14] and environmental factors [15,16] together with early non-motor symptoms, neurobehavioral disorders (for example depression, anxiety), cognitive impairment (dementia) and autonomic dysfunction (e.g., orthostasis and hyperhidrosis) [9,17,18] can provide possibilities for monitoring and early interventions. Levodopa-based preparations are still considered the treatment of choice; however, these agents are not able to slow or block disease progression and may generate dyskinesia [19]. Despite extensive research, no pharmacologically effective therapeutic is available to alter the progression of the disease. Investigation to discover and develop new treatment strategies [20,21] such as multi-modal application of gene therapy, stem cell therapy and immunomodulation is ongoing. A better understanding of PD pathogenesis (e.g., role of the gut-brain axis and microbiota [22,23]), chronology of neurodegeneration, identification of specific and sensitive biomarkers of PD and novel therapeutic targets [24], would facilitate improved PD therapies.

## Venoms for Parkinson’s disease

**Figure 1.**
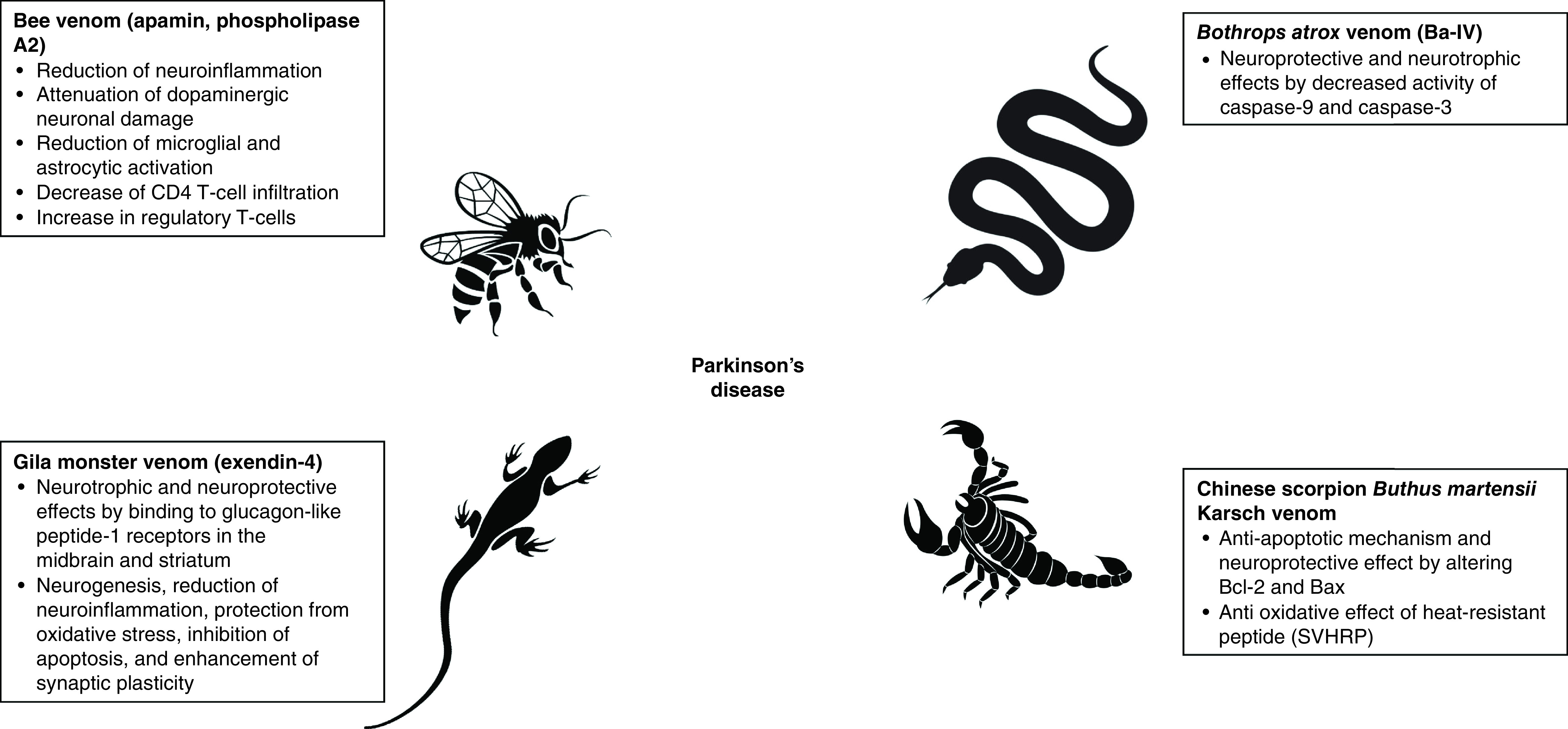
Proposed mechanisms of bee, lizard, scorpion and snake venoms as potential treatments for Parkinson’s disease.

Several disease-modifying candidates are currently under investigation. Venom-derived products [25,26], have offered a platform for development of novel medicines [27,28], some of which are already approved for clinical use [29,30]. Venoms from, for example, scorpions, snakes, spiders, bees, cone snails and sea anemones consist of a diverse range of proteins and peptides that target a broad spectrum of receptors in living organisms, for example, voltage-gated channels, ligand-gated channels, membrane transporters and enzymes [31,32]. Venoms have been investigated as treatments for cancer [29,30], pain [33,34], cardiovascular diseases, autoimmune diseases [28] and neurodegenerative disorders [25,26,35], including the focus of this special report, PD (Figure 1).

Bee venom includes peptides like melittin, apamin, mast cell degranulating peptide, adolapin, as well as enzymes such as phospholipase A2 (PLA2), hyaluronidase, amino acids and volatile compounds (such as isopentyl acetate and n-butyl acetate) [36,37] with a diverse range of pharmacological properties [35,38,39]. Substances from bee venom are proposed to treat PD [40], mainly based on their neuroprotective properties [41]. One of the underlying mechanisms in PD has been explained by chronic release of proinflammatory cytokines from activated astrocytes and microglia [42,43], where these molecules could exacerbate neurodegeneration in dopaminergic neurons [44]. Bee venom targets these inflammatory processes in PD [7] and reduces neuroinflammation. In 2011, Kim *et al.* [45] showed that bee venom injection (subcutaneously into an acupuncture point) in a mouse model of 1-methyl-4-phenyl-1,2,3,6-tetrahydropyridine (MPTP)-induced PD [46] could suppress neuroinflammation. Bee venom attenuates the MPTP-induced dopaminergic neuronal damage through blockade of phospho-Jun immunoreactivity [47]. Microglial activation is also reduced by bee venom as demonstrated by decreased levels of the microglial activation markers, MAC-1 and iNOS expression in the SNpc [45]. It has been proposed that suppression of proinflammatory molecules, such as cyclooxygenase-2, PLA2, TNF-α and IL-1 by bee venom exerts neuroprotective effects [45]. The administration of bee venom also reduces microglial activation and infiltration of CD4 T cells into the SNpc [48–50]. In addition, an increase in T-reg following the bee venom application has been suggested [48]. This hypothesis is supported by observations [51] that in the MPTP-treated mice, T-reg activation could block neuronal degeneration. A component of bee venom, PLA2, induces the expression of T-reg and consequently promotes dopaminergic neurons survival. PLA2 directly binds to the mannose receptor on dendritic cells and promotes PGE2 release, which results in T-reg differentiation in the MPTP model [49]. Bee venom, or PLA2 alone, improved motor coordination and balance in different models of PD [50], which suggests that PLA2 is the active pharmacological compound [52,53]. Another peptide from bee venom, apamin, has shown neuroprotective effects on mesencephalic or midbrain dopaminergic neurons in culture [54,55]. These findings suggest that several components of bee venom contribute to its pharmacological actions in neuroprotective effects in PD.

Studies suggest that efficacy of bee venom in PD is comparable to selective dopamine receptor agonists. For example, Anderson *et al.* [56] compared the neuroprotective effects of pramipexole (a potent and selective D3 dopamine receptor agonist) and bee venom in a subchronic MPTP mouse model of PD. Both treatments were equally effective against MPTP-induced damage to nigrostriatal dopamine neurons. Hyperactivation of astrocytes in SNpc in this model was also attenuated by bee venom and pramipexole, which contributed to a decrease in loss of dopaminergic neurons. These findings collectively highlight the role and importance of bee venom in neuroprotection or modulation of astrocytic activation [53].

Other animal models of PD have been used to investigate the effect of bee venom. In a rotenone model of PD in mice [57], bee venom improved locomotor behavior impairments and reversed the rotenone-related reduction in brain dopamine, serotonin, norepinephrine, GSH levels and paraoxonase activity and the significant elevation in brain malondialdehyde, TNF-α, IL-β, DNA damage and over-expression of caspase-3, Bax and Bcl-2 genes [57]. Based on these findings, the authors proposed that bee venom decreases neuroinflammation, oxidative stress and apoptosis [57]. These and several other positive preclinical results since 2007 [58–61] have encouraged scientists to test if bee venom or extracted compounds on individuals with neurodegenerative diseases [62].

A small number of clinical trials have been conducted in patients with PD. Cho *et al.* [63] investigated whether acupuncture and bee venom acupuncture would be beneficial for idiopathic PD. In this study, 43 patients with idiopathic PD were included and the Unified Parkinson’s Disease Rating Scale (UPDRS), the Parkinson’s Disease Quality of Life Questionnaire, the Beck Depression Inventory, the Berg Balance Scale and the time and number of steps required to walk 30 m were measured. Patients were treated twice per week for 8 weeks and findings showed that acupuncture and bee venom acupuncture similarly improved patients function in the tested parameters compared with the control group (did not receive any treatment) [63]. Doo *et al.* [64] assessed the combination of bee venom acupuncture with manual acupuncture, which turned out to be safe and improved motor function [64]. In a double-blinded, randomized controlled pilot study in 2016, potential disease-modifying characteristics of monthly bee venom injection in 40 patients with PD was investigated [65]. Monthly administration of bee venom for 11 months did not show any significant decrease in UPDRS (part III) scores in the ‘off’ condition, which was the primary outcome of this study [65]. In 2018, a triple-armed randomized controlled study [66] evaluated the efficacy of acupuncture and bee venom acupuncture twice a week for 12 weeks as an adjuvant therapy for idiopathic PD compared with a sham group. A total of 73 patients were recruited and treatment effects were investigated together with sustained therapeutic effect after completion of the study. Outcome measures were similar to the previous study by Cho *et al.* [63]. Both acupuncture and bee venom acupuncture showed significant improvement in function in the tested parameters that was maintained for bee venom acupuncture for 8 weeks after the end of therapy [66].

Larger sample sizes, higher doses and frequency, additional robust control groups, including proper primary and secondary outcomes must be considered for future PD clinical trials of bee venom [25].

Although bee venom has been most studied in this context, other venoms have also been tested for a potential therapeutic effect in PD, including scorpion venom [67,68], snake venom [69] and lizard venom [70–76]. Xu *et al.* [77] tested if the bioactive peptide derived from the scorpion venom (Chinese scorpion *Buthus martensii* Karsch) could alter apoptosis factors Bcl-2 and Bax in PD rats. In this study, 6-hydroxydopamine (6-OHDA) was used to induce an early PD rat model [78]. Findings showed that Bax increased significantly in the brain of 6-OHDA treated rats while Bcl-2 decreased significantly. In the venom-treated group, upregulation of Bax and down regulation of Bcl-2 were normalized. Based on the outcome, the authors proposed an anti-apoptotic mechanism contributed in the neuroprotective effect of the scorpion venom in this PD model [77]. Yin *et al.* [67] have also demonstrated that scorpion venom heat-resistant peptide (SVHRP), could protect oxidative stress in the 6-OHDA rat model [67]. Collectively, SVHRP seems to preserve the axonal function, promote upregulation of Bax and downregulation of Bcl-2 [67,77,79].

A snake venom, *Bothrops atrox* venom fraction Ba-IV, has been investigated as a potential candidate to treat PD [69]. In a cell model of PD (PC12 cells treated with 1-methyl-4-phenylpyridine, a dopaminergic neurotoxin), Ba-IV showed neuroprotective and neurotrophic effects. The underlying mechanism was linked to decreased activity of caspase-9 and caspase-3, which are apoptotic proteases and a potential neurotrophic effect [69].

Gila monster venom contains exendin-4, which is a 39-amino-acid peptide that binds to glucagon-like peptide-1 receptors, which are present in pancreatic β islet cells and in the midbrain and striatum. It has been shown that activation of neurons by exendin-4 leads to neurotrophic and neuroprotective effects, such as neurogenesis, reduction of neuroinflammation, protection from oxidative stress, inhibition of apoptosis and enhancement of synaptic plasticity [73]. A proof-of-concept, single-blind study with 45 moderate PD patients improved the Movement Disorder Society-Sponsored Revision of the Unified Parkinson’s Disease Rating Scale (MDS-UPDRS) score after 12 months of treatment with twice-daily injections of exendin-4 [75]. The effect was maintained for 12 months after the end of the treatment, which may suggest that this treatment is neuroprotective [76]. In a double-blind, placebo-controlled trial, 62 patients were treated with injections of exendin-4 every week, for 48 weeks and patients were followed for 12 weeks after the end of the treatment [74]. This study also showed that motor skills were improved in the treated group compared with the placebo group on the MDS-UPDRS score, for at least 12 weeks after the end of therapy [74]. Chen *et al.* [71] applied a sustained release formulation of exendin-4 (called PT302) in a rat model of 6-OHDA unilateral lesion. PT302 was found to exert a neuroprotective effect on the nigrostriatal dopaminergic neurons [71]. Taken together, current evidence supports the therapeutic potential of several venoms for PD [31].

## Conclusion & future perspective

It is undeniable that venoms are a rich source of bioactive compounds and current evidence supports the neuroprotective properties of some venoms [25]. Findings presented here highlight that venoms can slow down or even block neurodegeneration in PD. Despite this, only a few compounds have entered into clinical trial phases [63,80]. Translation issues from preclinic to clinic might be one of the reasons. Generally, sufficient scientific evidence is required first to justify human trials. Insufficient evidence stems from preclinical studies that remained preliminary, or have only been performed in one model with a lack of proper control groups or limited dose-response and time course of tests. Animal models to study neurodegenerative diseases have several limitations [81], in particular the current lack of understanding of the pathogenesis of PD. Since PD is a multifactorial complex disease, studying venoms effects in different animal models of PD is predicted to enhance success rate in translating results into humans. For example, animal models based on genetically modified processes, can only resemble a hereditary subtype of the disease. Many studies employ crude venom without evaluating individual components and this approach makes it difficult to identify specific neuroprotective mechanisms. Separation or isolation of a single biologically active fraction from the crude venom is methodologically challenging. Recombinant or biochemical production have been use to overcome this challenge, but selection of host and vector, or the synthesis process is not challenge free [82]. Chemical synthesis of proteins using solid-phase techniques is a fast and effective alternative technique with some limitations. It is just the matter of time until advancements in technology allow further investigation of the biologically active components of crude venoms.

Reports so far show no concerns about safety issues of venoms used in human trails of PD. A systematic review has mentioned one case of itching caused by the bee venom acupuncture [83]. However, in future trials, it is important to consider optimal dose for an effective and safe treatment and whether long-term treatment, based on long-term disease duration, would generate satisfactory results. In addition, one must be aware that venom side effects that include allergy, hemorrhage, necrosis or neurotoxicity might also occur in future human trials.

A recent review [84] has looked into 34 marine-derived natural compounds for PD therapy and five of these compounds have already entered into clinical trials. With regard to marine-derived venoms, α-Conotoxin from Conus textile, a selective inhibitor of nicotinic receptors was reported to increase dopamine release, might be a future potential candidate for treatment of PD [85]. Based on the history of successful drug development from other marine-derived venom [30], for example, ziconotide, derived from Conus magus venom and approved for severe chronic pain, it is not unlikely that there will be venom-based drugs for PD in the future.

**Executive summary**

- Age-related diseases, such as Parkinson’s disease (PD), pose a high negative impact on individuals, societies and healthcare systems.
- By 2040, up to 17 million people will be affected by PD, which requires focused planning to prevent or control its socioeconomic burden.
- Progressive dopaminergic neuron degeneration, α-synuclein protein aggregation, Lewy pathology, neuroinflammation, glia activation, mitochondrial dysfunction and oxidative stress have been proposed as underlying pathogenesis of PD.
- PD is characterized by motor dysfunction including bradykinesia, resting tremor and rigidity. Sensory alterations and neuropsychiatric disturbances are also common.
- Current therapy for PD is often partially effective and is associated with side effects; hence, disease-modifying therapies are under extensive investigation.
- Venoms from honey bees, snakes, scorpions and lizards have been investigated as disease modifiers of PD.
- Data from venom-based preclinical and clinical studies for PD are still limited; however, positive results, together with advancements in peptide technologies, may support their future use in therapy of PD.
